# Supplementary material for: Collaborative intelligence in AI: Evaluating the performance of a council of AIs on the USMLE
Source: PLOS Digit Health. 2025 Oct 9;4(10):e0000787. doi: 10.1371/journal.pdig.0000787 (PMC12510544; doi:10.1371/journal.pdig.0000787)
Supplement: S2 File — (ZIP) [file pdig.0000787.s002.zip › S2 Data Raw data files containing unprocessed question inputs and LLM outputs including transcripts of deliberations/STEP2_Question-98-1_Type-MC-NJ.docx]

**Facilitator** **[Round 1]:** Three days after an operation for uterine cancer, a hospitalized 67-year-old woman develops edema of the right lower extremity. Doppler ultrasonography shows a deep venous thrombosis (DVT). Review of the patient's record indicates that no DVT prophylaxis was initiated perioperatively. Which of the following is the best approach to prevent a similar adverse event in the future? (A) Conduct a nursing in-service regarding DVT prophylaxis (B) Develop an institutional policy with standardized orders for DVT prophylaxis (C) Encourage early ambulation during the postoperative period (D) Prescribe low-molecular-weight heparin for all postoperative patients (E) No intervention indicated because this is an isolated case and does not occur frequently

**AI_D:** RESTATING THE QUESTION: To paraphrase, we are considering a case where a 67-year-old woman who recently underwent a surgery for uterine cancer has developed edema in her right lower limb. The Doppler ultrasonography confirms the presence of a deep venous thrombosis (DVT). DVT prophylaxis was not provided around the time of operation. The question asks which action would be most effective in preventing this adverse event from recurring? These suggested actions include nursing education about DVT prophylaxis, institutional policy for DVT prophylaxis, early postoperative mobilization, prescription of low-molecular-weight heparin for all postoperative patients, or no intervention considering the event isolated and infrequent. PLANNING: Here, the assumptions made include the role of each of these suggested interventions in preventing postoperative DVT. Each of these options need to be evaluated considering the benefits and possible drawbacks. The crucial assumption that needs prioritization is the standardization of care with a focus on preventive measures, in this case, DVT prophylaxis. CLINICAL REASONING: Postoperative DVT is a recognized risk and measures for its prevention are integral to patient safety. Although early ambulation and prescription of low-molecular-weight heparin could help prevent DVT, these are individual patient-based interventions. Likewise, a nursing in-service would educate staff but might not ensure standardization of care. However, developing an institutional policy with standardized orders for DVT prophylaxis would ensure that every patient receives consistent care, regardless of individual practices or variables. It would institutionalize the prevention of such an adverse event. CONCLUDING: It can therefore be concluded that a system-level change, namely the development of institutional policy with standardized DVT prophylaxis, could potentially have the greatest impact on preventing such events in the future, as it would ensure consistent and proactive care for all postoperative patients. RESPONSE: So, the answer to the question, "Which of the following is the best approach to prevent a similar adverse event in the future?" is (B) Develop an institutional policy with standardized orders for DVT prophylaxis.

**AI_C:** RESTATING THE QUESTION: The case revolves around a 67-year-old woman who, following a uterine cancer surgery, has developed deep venous thrombosis (DVT), a condition characterized by the formation of blood clots within the deep veins of the body, usually in the lower limbs. No preventative measures against DVT were initiated before surgery. It raises the question, what measures can be taken to prevent the recurrence of a similar situation in the future? The options include conducting a nursing course about DVT prevention, instituting standardized DVT prophylaxis procedures, encouraging early post-surgery mobility, prescribing low-molecular-weight heparin for all post-surgery patients, or considering it an isolated case needing no particular intervention. PLANNING: There are several assumptions here: (1) the DVT resulted from the absence of perioperative DVT prophylaxis, (2) an institutional policy would ensure DVT prophylaxis adherence, (3) early ambulation helps prevent DVT, (4) low-molecular-weight heparin as a standard prescription for all postoperative patients would reduce DVT incidence, and (5) this is a one-off situation not warranting further action. By dissecting these assumptions, we deduce that the heart of this scenario is the hospital's protocol (or lack thereof) for preventing DVT in postoperative patients. The optimal approach would prioritize addressing this systemic issue. CLINICAL REASONING: Looking clinically at this case, it's clear that the absence or inadequacy of the DVT prophylaxis protocol in the hospital likely contributed to this patient's condition. While nursing education, individual prescribing practices, and patient habits can play roles in preventing DVT, these are more individual-based and less consistent than a solid institutional policy mandating DVT prophylaxis. Such a policy would standardize care and help reduce the incidence of DVT in postoperative patients, with the added benefit of avoiding isolated and infrequent but significant adverse events. CONCLUDING: In conclusion, based on my knowledge and clinical reasoning, the most effective way to curb the incidence of events like this in the future is to change the institution's policy to include mandatory standardized DVT prophylaxis orders. This systemic approach would ensure that all patients get the care needed to prevent such a complication. RESPONSE: So, the best approach to prevent similar adverse events in the future would be to (B) Develop an institutional policy with standardized orders for DVT prophylaxis.

**AI_E:** (1) RESTATING THE QUESTION: The query here is asking for the most appropriate preventive measure to avoid recurrences of deep venous thrombosis (DVT) in a hospital scenario, particularly in postoperative patients. DVT is a condition where a blood clot forms in the deep veins of the body, usually the legs or thighs. This happened to a woman who had no DVT prophylaxis administered during her perioperative period. (2) PLANNING: An assumption that can be made from the question is that there was either an oversight in providing DVT prophylaxis to this patient, or possibly, there were no set standards for DVT prophylaxis in the institution. This can be questioned; should a system of standardized orders be implemented? If DVT prophylaxis had been provided, the risk of DVT might have been mitigated. However, DVT can occur despite prophylaxis, so implementing it as a treatment for everyone may not be necessary. The focus should instead be on identifying patients at high risk for DVT - such as postoperative patients - and ensuring they receive optimal prevention. (3) CLINICAL REASONING: In this clinical scenario, the woman developed DVT due to a combination of factors, including her recent surgical procedure, possible immobility, and the absence of preventive measures during the perioperative period. The presence of multiple risk factors amplifies the risk. Establishing an institutional policy with standardized orders for DVT prophylaxis provides a comprehensive approach to this issue. (4) CONCLUDING: Based on a sequential clinical thought process, the best approach lies in preventing DVT through standardized prophylaxis orders across the institution. This systematic effort will ensure identification of high-risk patients and prompt administration of prophylaxis. (5) RESPONSE: Thus, the most effective strategy to avert a similar adverse event in the future would be to (B) Develop an institutional policy with standardized orders for DVT prophylaxis. This approach ensures that all high-risk patients, such as post-surgical candidates like the 67-year-old woman in this case, receive timely and appropriate DVT prevention.

**AI_B:** (1) RESTATING THE QUESTION: The scenario describes the case of a 67-year-old lady who underwent a surgical procedure for uterine cancer. She developed edema in her right lower limb three days after her operation, diagnosed as deep venous thrombosis (DVT) via Doppler ultrasonography. It is noted that no prophylactic measures were taken perioperatively to avoid DVT. The question at hand is about the best preventive method to avoid similar adverse events in the future. Options include conducting an in-service for nurses about DVT prophylaxis, developing a standardized policy for DVT prophylaxis, encouraging patients to ambulate early after surgery, routinely prescribing low-molecular-weight heparin (a kind of anticoagulant) to postoperative patients, or considering the incident as an isolated case and not making any intervention. (2) PLANNING: We must assume that DVT is not an isolated event after operative procedures, especially in cancer patients and elderly ones, and measures to prevent it should be in place. Additionally, not all patients may need pharmacological prophylaxis like low-molecular-weight heparin. The main alternate assumption is a need for a structured proactive approach towards DVT prophylaxis which would be most effective in averting such occurrences in the future. (3) CLINICAL REASONING: Surgical procedures, especially those involving cancer and performed on elderly individuals, pose a significant risk for the development of DVT due to hypercoagulability, venous stasis, and endothelial injury (Virchow's triad). Early ambulation postoperatively is beneficial but might not be possible in all patients and doesn't eliminate the DVT risk entirely. Pharmacological prophylaxis should be individualized rather than a blanket prescription for all postoperative patients, considering the risk of bleeding complications. Providing nurses with inservice education is valuable, but doesn't guarantee consistent prophylaxis across the board. (4) CONCLUDING: Thus, an institutional policy with standardized DVT prophylaxis orders emerges as the best option. This ensures every patient is evaluated for DVT risk, and necessary preventive steps are taken as per the guideline. This method reduces variability in practice and helps prevent DVT-associated morbidity and mortality in postoperative patients. (5) RESPONSE: Therefore, the best approach to avoid similar adverse events like DVT after surgery in the future would be to (B) Develop an institutional policy with standardized orders for DVT prophylaxis. This ensures that all patients undergo appropriate perioperative DVT risk evaluation, and appropriate measures are initiated to reduce this risk.

**AI_A:** (1) RESTATING THE QUESTION: This question revolves around a medical scenario wherein a senior woman patient has unfortunately developed deep venous thrombosis (DVT), a blood clot in a deep vein usually in the legs, post her operation for uterine cancer. We are asked to evaluate the best future course of action to prevent another occurrence of this adverse event. DVT prophylaxis, which has been neglected here, is a preventive measure to reduce the risk of DVT formation. Various approaches to this problem have been suggested and an evaluation is asked for. (2) PLANNING: Let's evaluate the options provided: (A) The first is educating nurses about DVT prophylaxis, which may ensure that the nursing staff is fully informed on the topic, can recognize the risk factors and may insist on prevention methods. (B) Secondly, developing an institutional policy and standardized order for DVT prophylaxis takes a universal approach, assuming all patients may be at risk, which might increase the chances of preventing DVT in all applicable cases. (C) Encouraging early postoperative movement is an integral part of DVT prevention. However, it is not the only measure and may not always be applicable to all patients. (D) Prescribing low-molecular-weight heparin, a blood thinner, to all postoperative patients considers all postops to be at risk by default. But this disregards individual patient responses or contraindications. (E) Lastly, the option of no intervention, assuming this to be an isolated case. But given the known DVT risks after surgeries, particularly in elderly cancer-patients post-operation, the logic behind ignoring the issue seems flawed. Judging from these, an integrated approach involving both institutional policy (B) and informed nursing staff (A), used in tandem could practically offer the most comprehensive coverage. (3) CLINICAL REASONING: If we consider option A, while educating nursing staff about DVT prophylaxis is important, it doesn't guarantee its application to every at-risk patient. Option B on the other hand, if a standardized policy for DVT prophylaxis is developed and implemented, it will automatically involve prescribing prophylaxis to those patients who are considered to be at risk of DVT. This approach is more systematic and ensures that no at-risk patient will miss out on the prevention unless contraindicated. With regard to C, movements are important in preventing DVT but they are not possible for every patient due to different recovery postures from operations, and not sufficient independently to prevent DVT. Option D is not always possible as it disregards patient-specific contraindications to blood thinners like severe bleeding risk etc. For E, assuming it's an isolated case leads to ignoring a potential risk and setting a dangerous trend in patient care. (4) CONCLUDING: It can be concluded that broad-based systemic measures coupled with personnel awareness could offer the most comprehensive protection. Remember, prescriptive methods might seem exhaustive but sometimes they fail to accommodate the unique needs and risk factors of individual patients. (5) RESPONSE: Hence, to ensure the future prevention of similar adverse events, the best approach would be to 'develop an institutional policy with standardized orders for DVT prophylaxis' (B). This would ensure a well-structured, comprehensive, and, most importantly, patient-focused approach to DVT prevention.

**Facilitator** The consensus statement synthesizes the responses of AI_A, AI_B, AI_C, AI_D, and AI_E as follows: The question revolves around a 67-year-old woman who developed deep venous thrombosis (DVT) postoperatively after a uterine cancer surgery. The absence of DVT prophylaxis during the perioperative period is noted. The task is to identify the most effective approach to prevent a similar adverse event in the future. The options provided include educating nurses about DVT prophylaxis, developing an institutional policy with standardized orders for DVT prophylaxis, encouraging early postoperative movement, prescribing low-molecular-weight heparin to all postoperative patients, or considering this an isolated case and not intervening. The consensus among the AI responses is that the best approach to prevent a similar adverse event in the future is to develop an institutional policy with standardized orders for DVT prophylaxis. This approach ensures a systematic and comprehensive coverage for DVT prevention, reducing variability in practice and ensuring that all patients are evaluated for DVT risk. It also ensures that necessary preventive steps are taken as per the guideline, providing consistent and proactive care for all postoperative patients. While educating nursing staff, encouraging early postoperative movement, and prescribing low-molecular-weight heparin can contribute to DVT prevention, these measures are not sufficient independently and may not be applicable to all patients. Furthermore, considering this an isolated case and not intervening could lead to ignoring a potential risk and setting a dangerous trend in patient care. Therefore, the consensus is that the best approach to prevent a similar adverse event in the future is to develop an institutional policy with standardized orders for DVT prophylaxis. This approach ensures that all high-risk patients, such as post-surgical candidates, receive timely and appropriate DVT prevention.
